# Metabolic profilings of rat INS-1 β-cells under changing levels of essential amino acids

**DOI:** 10.1038/s41597-022-01436-w

**Published:** 2022-06-14

**Authors:** Lianbin Xu, Xueyan Lin, Xiuli Li, Zhiyong Hu, Qiuling Hou, Yun Wang, Zhonghua Wang

**Affiliations:** 1 College of Animal Science and Technology, Shandong Agricultural University, Taian, 271018 P. R. China; 2 Institute of Animal Immune Engineering, Jiangsu Academy of Agricultural Sciences, Nanjing, 210014 P. R. China

**Keywords:** Metabolomics, High-throughput screening, Diabetes complications

## Abstract

Application of mass spectrometry enables the detection of metabolic differences between organisms with different nutritional settings. Divergence in the metabolic fingerprints of rat pancreatic INS-1 β-cells were systematically captured with regard to ten individual essential amino acid (EAA) availability. A high-resolution tandem mass spectrometry system coupled to liquid chromatography produced a horizontal comparison of metabolic profilings of β-cells with individual EAA elevated to 10 mmol/L by turn or removal individual EAA from the medium one by one. Quality control samples were injected at regular intervals throughout the analytical run to monitor and evaluate the stability of the system. The raw data of samples and reference compounds including study protocols have been deposited in the open metabolomics database MetaboLights to enable efficient reuse of the datasets, such as investigating the difference in metabolic process between diverse EAAs as well as screening and verifying potential metabolites affecting insulin secretion and β-cell function.

## Abstract (summary)

**Table:** 

Measurement(s)	metabolomic profiling
Technology Type(s)	Ultra High-performance Liquid Chromatography/Tandem Mass Spectrometry
Factor Type(s)	arginine • histidine • isoleucine • leucine • lysine • methionine • phenylalanine • threonine • tryptophan • valine
Sample Characteristic - Organism	Rattus norvegicus

## Background & Summary

Homoeostatic regulation of fuel metabolism in the body is a tightly controlled process and dysregulation can result in pathological conditions such as diabetes mellitus^1^, which is a major threat to the lives and well-being of individuals in the world. Amino acids (AAs), as the third major class of nutrients following carbohydrates and lipids, play critical roles in pancreatic β-cell function and insulin secretion^1^. Ample evidence indicated that several AAs could elicit positive and/or negative effects on β-cell insulin release both *in vitro* and *in vivo*, which is mostly dependent on the AA type, duration of exposure and concentration^2^. However, systematic comparison of metabolic processes between different individual essential AAs (EAAs) in β-cell has not been well characterized.

It is generally accepted that AAs regulate both the triggering and amplification pathways of insulin secretion partly through acting as a substrate for the TCA cycle and/or redox shuttles with subsequent generation of ATP^3^. Glucose-stimulated insulin secretion (GSIS) requires that this sugar is metabolized and that the coupling of its metabolism to secretion involves a variety of critical intermediates and co-factors, which can also be produced by several AAs metabolism^4^. In addition, special metabolite such as nitric oxide generated by arginine may induce endoplasmic reticulum stress, damage β-cell function and finally reduced the insulin export^2,5^. These findings suggested that AAs as well as their metabolic products have significant effects on insulin release. Therefore, capture of metabolic snapshots in the β-cells exposed by individual EAA provides an effective way to understand the biochemical mechanisms underlying the coupling of stimulation and insulin secretion.

AA administered alone at physiological concentrations do not modulate GSIS, but supplied in specific combinations at physiological concentrations or individually at elevated concentrations can elevate GSIS^2,6^. Previous work showed that a concentration of 10 mmol/L AA provided a robust and reproducible stimulus to insulin secretion and has been used in several experiments^7–9^. In this study, we comprehensively compared the metabolic profilings of β-cells obtained from 130 samples with different individual EAA availability (0 or 10 mmol/L for each EAA) based on a liquid chromatography coupled to tandem mass spectrometry (LC-MS/MS). All data have been deposited in the MetaboLights repository^10^.

By making this dataset publically available, it lends itself to applications in the biochemistry of insulin secretion, metabolic cross-study comparisons or investigations into reproducibility of data analysis in metabolomics. Considering the horizontal study design, this experimental dataset allows for diverse utilizations. First, these data gave a comprehensive description of different EAA metabolic profilings in β-cells under two nutritional settings, which helps to investigate the difference in metabolic process between individual EAAs. Also, it may assist researchers in studying the underlying molecular mechanisms of EAA affecting β-cell function and insulin secretion. Moreover, data can be more broadly used for illustration of the coupling between AA metabolism and insulin release, which contributes to screen key metabolites and co-factors with potential therapeutic values.

## Methods

### Study design

The rat pancreatic INS-1 β-cells used in this study were kindly provided by Professor Ling Gao in Scientific Center, Shandong Provincial Hospital affiliated to Shandong University. Pancreatic β-cells (24 passages) were grown and maintained in RPMI-1640 medium (Gibco, NY, USA) containing 11.1 mmol/L glucose with 10% (v/v) fetal bovine serum (FBS; Gibco, NY, USA), 10 mmo/L N’-a-hydroxythylpiperazine-N’-ethanesulfanic acid (HEPES; Solarbio, Beijing, China), 1 mmol/L sodium pyruvate (Solarbio, Beijing, China), 50 μmol/L β-mercaptoethanol (Sigma-Aldrich, MO, USA), 100 IU/mL penicillin (Sigma-Aldrich, MO, USA) and 100 μg/mL streptomycin (Sigma-Aldrich, MO, USA) at 37 °C in a humidified atmosphere of 5% CO_2_.

**Fig. 1:**
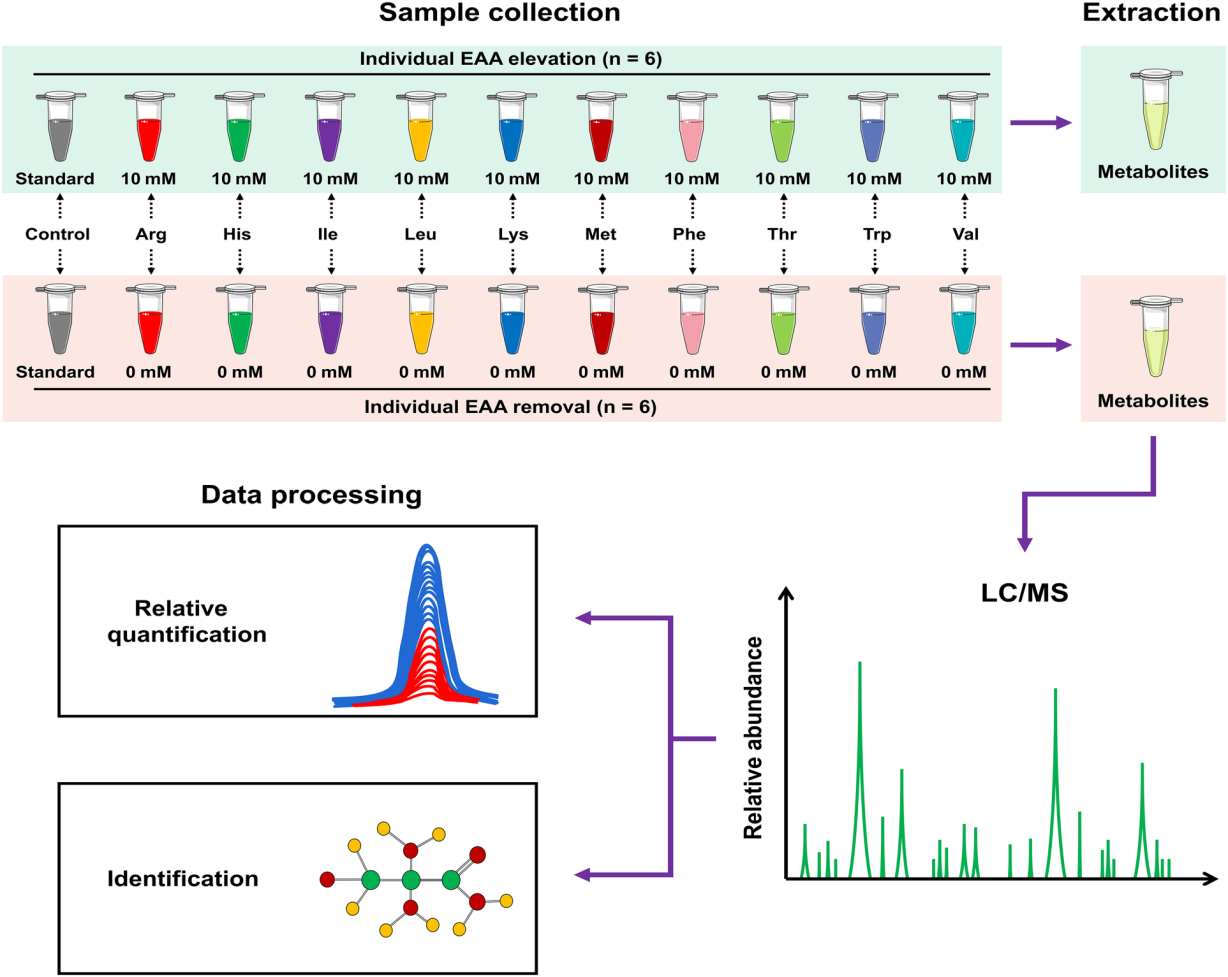
Overview of the experimental workflow. Samples were collected from INS-1 β-cells with different individual essential amino acid treatments and then extracted for metabolomics analyses using a high-resolution tandem mass spectrometry system coupled to liquid chromatography (*n* = 6). Data were pre-processed, including data quality checks, data normalization and relative quantification and identification of metabolites.

The trial design and analytical process were presented in Fig. 1. In order to investigate the chronic metabolic responses to various EAA stimulations, INS-1 β-cells were seeded into culture dishes at a proper density, and allowed to adhere to the dishes overnight. After that, the medium was removed, and the cells were randomly assigned to one of the ten paired treatments where the concentration of special one EAA was elevated to 10 mmol/L or was decreased to 0 mmol/L (Control, + Arg/-Arg, + His/-His, + Ile/-Ile, + Leu/-Leu, + Lys/-Lys, + Met/-Met, + Phe/-Phe, + Thr/-Thr, + Trp/-Trp and + Val/-Val, respectively). After 24-h treatment, all cell samples were taken at the same time, frozen in liquid nitrogen, and stored at −80 °C until required.

### Sample preparation

Cell samples were subjected to an untargeted metabolite analysis by LC-MS/MS of polar extracts. Total metabolites were extracted from each group using approximately 1 × 10^7^ cells in 1 mL of methanolic acetonitrile aqueous solution (2:2:1, v/v/v). The cells were then vortexed for 60 s, ultrasonicated for 30 min at 4 °C twice, and then kept at −20 °C for 1 h to precipitate protein. The solution was centrifuged at 14,000 rcf for 20 min at 4 °C, and then the supernatant was stored at −80 °C until future use.

### Chromatography

The details of the operating procedures have been described in the previous study^11^. Briefly, INS-1 cell samples were separated on an Agilent 1290 Infinity LC Ultra-High-Performance Liquid Chromatography System (Agilent Technologies, CA, USA) using a HILIC column. The column temperature was 25 °C, and the flow rate was 0.3 mL/min. The mobile phase of composition A consists of water, 25 mmol/L ammonium acetate and 25 mmol/L ammonia, and B was acetonitrile. The gradient elution procedure was as follows: 95% B (0–1 min); B varied linearly from 95% to 65% (1–14 min), B decreased linearly from 65% to 40% (14–16 min); B was maintained at 40% (16–18 min), B increased linearly from 40% to 95% (18–18.1 min), and finally B was maintained at 95% (18.1–23 min). The sample was placed in a 4 °C autosampler. In order to avoid the effects of instrument detection signal fluctuations, continuous analysis of samples was performed randomly. Pooled quality control (QC) samples (generated by taking an equal aliquot of all the samples included in the experiment) were run three times before the beginning of the sample queue forcolumn conditioning and every ten injections thereafter to assess inconsistencies that are particularly evident in large batch acquisitions in terms of retention time drifts and variation in ion intensity over time.

### Mass spectrometry

Electrospray ionization (ESI) positive and negative ion modes were used for detection. The sample was separated by UHPLC and subjected to mass spectrometry using a TripleTOF 5600 mass spectrometer (AB SCIEX, Framingham, MA, USA). The ESI source conditions after HILIC chromatographic separation were as follows: ion source gas 1 (gas 1: nitrogen): 60; ion source gas 2 (gas 2: nitrogen): 60; curtain gas (CUR): 30; source temperature: 600 °C; ion spray voltage floating (ISVF) ± 5500 V (positive and negative modes); TOF MS scan m/z range: approximately 60–1000 Da; product ion scan m/z range: approximately 25–1000 Da; TOF MS scan accumulation time 0.20 s/spectra; and product ion scan accumulation time 0.05 s/spectra. Secondary MS was obtained using information-dependent acquisition (IDA) and high sensitivity mode, with a declustering potential (DP): ± 60 V (both positive and negative modes), collision energy: 35 ± 15 eV. The IDA settings were as follows: exclude isotopes within 4 Da; and candidate ions to monitor per cycle: 6.

### Data processing and transformation

**Fig. 2:**
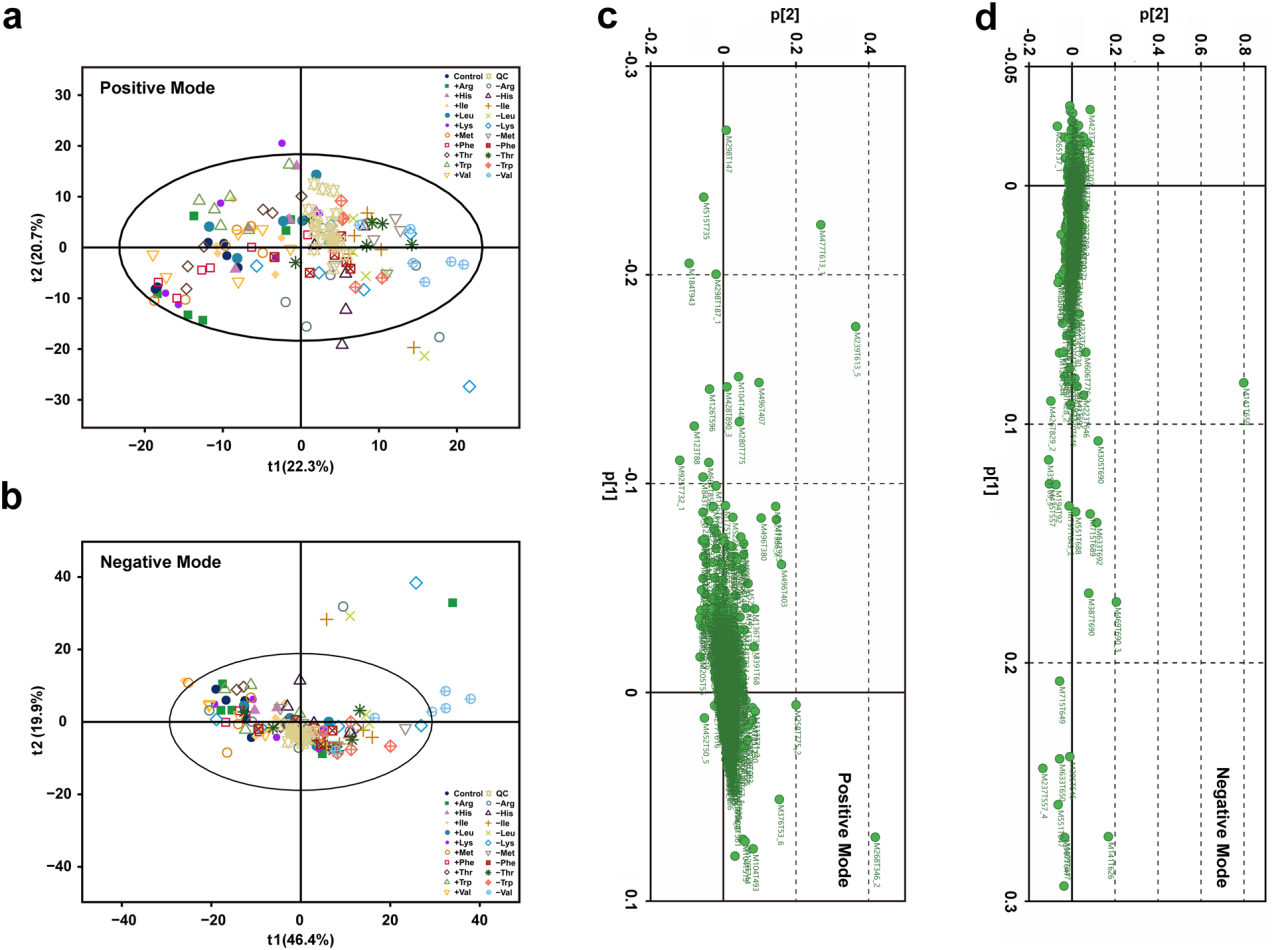
Principal component analysis of the data sets acquired in positive and negative ion modes (**a** and **b**) and the loadings for the metabolites (**c** and **d**). The first two principal components are shown. Total signal normalization and Pareto scaling were applied to both data sets. Quality controls, mixed of each group samples, cluster distinctively outside the main group.

Non-targeted LC-MS/MS vendor raw data files were converted to the open source format mzML using the program ProteoWizard^12^, and then the XCMS program was used for peak alignment, retention time correction, and peak area extraction^13^. Metabolite structure identification used a method of accurate mass matching (<25 ppm) and secondary spectral matched against in-house tandem MS spectral library (Shanghai Applied Protein Technology, Shanghai, China). The in-house database has >30 thousand substances, which contains 6000 + metabolites (4000 + from human and animals, 2000 + from plants) identified by reference materials. And other 24000 + metabolites were from 4 public database: Mass Bank, Metlin, HMDB, the secondary standard spectrum library which corresponds to MoNA. The identification level of the in-house database is level 2 and above^14^. Overall, The MS/MS spectra matching score was calculated using dot-product algorithm, which take the fragments and intensities into consideration. The matching score cutoff was set as 0.8, and the MS/MS spectra matching results were confirmed with standards. In the extracted ion features, only the variables having >2/3 of the nonzero measurement values in at least one group were kept retained. To make the metabolomics data reproducible, the relative standard derivation (RSD) of the peaks in the QC samples larger than 30% were filtered out. Pattern recognition was performed with the application of SIMCA-P 14.1 software (Umetrics, Umea, Sweden). The data were preprocessed by Pareto-scaling for unsupervised principal component analysis (PCA, Fig. 2a,b), and the total features analyzed in positive and netative mode were consistent. The loadings for the metabolites were shown in Fig. 2c,d. Statistical differences were performed by one-way ANOVA for multiple comparisons among three concentrations of each individual EAA. Adjusted *P* value (*Q* value) was used to decrease the false discovery rate by the method of Benjamini-Hochberg. Metabolites with *Q* value < 0.05 were selected as metabolites with significant differences.

## Data Records

The raw LC-MS/MS data files for individual EAA administrations were deposited at the MetaboLights^10^ database (http://www.ebi.ac.uk/metabolights) of the European Bioinformatics Institute under MTBLS3963^15^. Each MetaboLights entry contains protocols about sample collection, extraction, chromatography, mass spectrometry, metabolite identification, and data transformation.

## Technical Validation

In mass spectrometry experiments, acquired data needs to be checked for unintended systematic and random factors that may distort analysis. Thus, different strategies were used to assure data quality. First, randomization orders were created and followed for sample extraction, LC-MS derivatization, and MS run orders. Second, QCs consisting of mixed samples were injected at regular intervals throughout the analytical run to monitor and evaluate the stability of the system and the reliability of the experimental data. The stability of the experimental system was further inspected using PCA on the processed sample by feature matrices, acquired in positive and negative ion modes. PCA is a well-established technique that can be used to provide a first overview of the data with regard to questions related to high variance, and sample clusters, trends, and outliers^16^. As shown in Fig. 2a,b, QC samples cluster distinctively, suggesting absence of significant non-biological induced variation in the experiment.

## Data Availability

The acquired metabolite profiles were processed by using ProteoWizard package (http://proteowizard.sourceforge.net), XCMS Online software (https://xcmsonline.scripps.edu/), SIMCA 13.0 (Umetrics AB, Umea, Sweden) software and MetaboAnalyst plotform (https://www.metaboanalyst.ca), respectively.
